# GAUGING INTEREST AND NEEDS IN PROFESSIONAL DEVELOPMENT AND CONTINUING EDUCATION IN AGING IN MANITOBA, CANADA

**DOI:** 10.1093/geroni/igac059.3066

**Published:** 2022-12-20

**Authors:** Michelle Porter, William Kops, Nicole Dunn

**Affiliations:** University of Manitoba, Winnipeg, Manitoba, Canada; University of Manitoba, Winnipeg, Manitoba, Canada; University of Manitoba, Winnipeg, Manitoba, Canada

## Abstract

The Centre on Aging at the University of Manitoba in Canada conducted a needs assessment on continuing education (CE) and professional development (PD) in aging with people who work and connect with older adults. During winter and spring 2022, an online survey of 35 questions asked respondents about their own or their staff and volunteer topics of interest, as well as logistics of CE/PD workshop training. A total of 146 participants responded. Respondents were from different regions of Manitoba, with the highest proportion (42%) located in the Winnipeg region. About 86% were female and 12% male, ranging in age from 18 to 75: with most falling in the 35 to 64 age range. Respondents were asked to indicate their level of interest for topics on a five-point scale from Not Interested to Very Interested. The topics Aging Through the Lifespan and Wellness received the highest number of responses, while Indigenous Aging received the least. Across the 1,712 individual ratings of suggested topics, the highest proportion were rated as interested (35%) and very interested (31%); while 19% rated level of interest as moderate, 11% minimal, and 4% not interested. When asked about potential workshop formats, the majority (63%) preferred a mix of both in-person and online delivery formats with preference for 1–2-hour workshops and a certificate as proof of completion. About two-thirds indicated a requirement to complete CE/PD credits to maintain related credentials. These findings provide considerations for higher education institutions on CE/PD for those working and connecting with older adults.

